# Economic evaluation of a multimorbidity patient centered care model implemented in the Chilean public health system

**DOI:** 10.1186/s12913-023-09970-y

**Published:** 2023-09-29

**Authors:** Paula Zamorano, Manuel Antonio Espinoza, Teresita Varela, Tomas Abbott, Alvaro Tellez, Nicolás Armijo, Francisco Suarez

**Affiliations:** 1 Centro de Innovación en Salud ANCORA UC, Facultad de Medicina, Pontificia Universidad Católica de Chile, Santiago, Chile; 2 Health Technology Assessment Unit, Center of Clinical Research, Pontificia Universidad Católica de Chile, Santiago, Chile; 3 Department of Public health, Pontificia Universidad Católica de Chile, Santiago, Chile; 4 Department of Family Medicine, Pontificia Universidad Católica de Chile, Santiago, Chile; 5 Unidad de Análisis y Gestión de la información, Servicio de Salud Metropolitano Sur Oriente, Santiago, Chile

**Keywords:** Multimorbidity, Economic evaluation, Implementation science, Transactional analysis, Chile

## Abstract

Multimorbidity and patient-centered care approaches are growing challenges for health systems and patients. The cost of multimorbidity patients and the transition to a new care strategy is still sightly explored. In Chile, more than 70% of the adult population suffer from multimorbidity, opening an opportunity to implement a Multimorbidity patient-centered care model. The objective of this study was to perform an economic evaluation of the model from the public health system perspective.

The methodology used a cost-consequence evaluation comparing seven exposed with seven unexposed primary care centers, and their reference hospitals. It followed three steps. First, we performed a Time-Driven Activity-Based Costing with routinely collected data routinely collected. Second, we run a comparative analysis through a propensity score matching and an estimation of the attributable costs to health services utilization at primary, secondary and tertiary care and health outcomes. Third, we estimated implementation and transaction costs.

Results showed savings in aggregate costs of the total population (-0.12 (0.03) p < 0.01) during the period under evaluation. Costs in primary care showed a significant increase, whereas tertiary care showed significant savings. Health outcomes were associated with higher survival in patients under the new care model (HR 0.70 (0.05) p < 0.01). Implementation and transaction costs increased as the number of pilot intervention centers increased, and they represented 0,07% of the total annual budget of the Servicio de Salud Metropolitano Sur Oriente. After three years of piloting, the implementation and transaction cost for the total period was USD 1,838,767 and 393,775, respectively.

The study’s findings confirm the purpose of the new model to place primary health care at the center of care for people with non-communicable chronic diseases. Thus, it is necessary to consider implementation and transaction costs to introduce a broad health system multimorbidity approach. The health system should assume some of them permanently to guarantee sustainability and facilitate scale-up.

**Supplementary Information:**

The online version contains supplementary material available at 10.1186/s12913-023-09970-y.

## Introduction

Multimorbidity (MM), defined as two or more chronic conditions in the same person [1, 2], is positioned as a public health problem worldwide, with a prevalence of 43% in the adult population of Latin America and the Caribbean [3]. MM negatively impacts patients, impairing functionality and quality of life, determining polypharmacy and adverse events, increasing mental health problems and mortality [4, 5]. In addition, the health system increases costs due to the greater use of health services, including more emergency room consultations and extended hospital stays [6, 7]. At the population level, MM appears relatively earlier in less advantaged socioeconomic groups, which deepens health inequalities [8]. Health systems should look for innovative interventions to tackle this problem in this context.

The evidence indicates that healthcare should adopt a patient-centered care model [9–11]. In this model the health system reorganizes roles and activities to meet patients’ needs, values, and preferences. Piloted interventions which fall under this type of model have shown effectiveness. This is the case of the case-management model [12, 13], disease management and self-management [14, 15], continuity of care [16] and risk stratification [17]. Theoretical frameworks, action plans for implementation and transferability have been developed mainly in Europe [18, 19]. Nevertheless, their description is rather general, leaving aside the implementation process in real practice and the associated costs.

Shifting toward MM approach involves significant costs at all levels of healthcare. Studies usually present annual cost estimates per patient with MM based on a diagnostic group and mainly from medical services [7]. However, given the risk stratification proposed by the model, cost estimates should consider high, medium, and low complexity costs. In addition to direct clinical costs, the health system incurs implementation and transaction expenses, which are rarely reported in costing studies [4], even though they are essential for jurisdictions planning to implement this change of care model.

In Chile, 70,6% of adults have multimorbidity [20]. The health system’s main adults chronic care is based on a single diagnostic approach [21–24] where its capacity can offer care to a maximum of approximately 4 million chronically ill patients out of 11 million patients with chronic conditions [20]. This model carries the risk of using the system’s capacities inefficiently, i.e. deriving resources to people with relatively lower needs than others. In response, a Multimorbidity patient-centered care model (MPCM) [25, 26] was implemented by the Centro de Innovacion en Salud ANCORA UC (CISAUC), National Health Fund (FONASA) with the Servicio Metropolitano Sur Oriente (SSMSO) as a pilot study between 2017 and 2020. The objective was to prevent complications from MM and thus the demand for secondary and tertiary care through reorganizing existing chronic services towards patient-centered care based on risk stratification, case management, self-management, shared responsibility, and continuity of care.

The MPCM responds to one of the strategic objectives of action for controlling non-communicable chronic diseases in the Americas 2013–2019 developed by Pan American Health Organization [27] and has already reported implementation and performance results showing positive results in terms of indicators of health system performance and health outcomes [28–31]. However, the impact on direct, implementation and transaction costs has yet to be evaluated in the Chilean context. Although interventions that produce better outcomes often require additional resources, this particular intervention may generate savings in several items. In this context, whether the MCMP is more, equal, or less costly for the system is an empirical question. The objective of this study was to perform an economic evaluation of the model from the public health system perspective.

## Methods

We performed a prospective non-concurrent study to estimate the effect of the MPCM model on healthcare costs and patients’ expected survival using routinely collected data of the the health system. The evaluation compared 20,359 patients treated in seven pilot centers where MPCM was implemented versus 26,340 patients receiving standard care in seven control centers from April 2017 to December 2019. The intervention began with four primary health care centers (PHC) and expanded to seven exposed PHC in 2018. Groups were analyzed simultaneously during the intervention period. Control centers were selected by territorial proximity and the amount of coverage population to have similar populations and PHC. Other descriptive variables were blinded to the researchers. Of the total sample, 5,706 (21%) control patients reported no costs despite having records of chronic disease and multimorbidity risk stratification. These patients probably continued their health care outside the public health system limiting the capture of data. We explored the effect of including or excluding this patient on the results, and we found that their exclusion produced only marginal differences and did not affect the main interpretations. Hence, the analysis was performed for the total population and those who reported costs.

### Direct costs

Patient-level direct costs were estimated using Time-Driven Activity-Based Costing (TDABC), which provides an accurate and realistic costing compared to standard costs [32]. We used a database provided by the Unidad de Gestión y Análisis de la Información en Salud at SSMSO, which collected the routine health records. We estimated the cost rate per minute (resource generator) of each clinical activity performed with patients (drivers). The activities included primary care (all health professional clinical consultants, medical emergency consultants and drugs dispense), secondary care (all specialist physician consultants, drug dispense and ambulatory surgery) and tertiary (all medical emergency room consultations, all hospitalization and surgery) care. Outpatient procedures at the primary and secondary levels were excluded. Then, we estimated the time (in minutes) every patient uses for every activity. These two parameters allow us to estimate the expected cost of each activity, and the sum of them in one year, the annual expected cost in one patient. This individual-based costing data was used for the comparative analysis between control and MPCM arms.

We evaluated the effect of MPCM on two outcomes, direct costs attributable to the use of health care services (at primary, secondary, and tertiary levels) according to their ACG risk (high risk = ACG risk 4–5; moderate and low risk = ACG 1,2 and 3) [33, 34] and overall survival for the total population. To minimize the selection bias of the sample, analyses were performed using the propensity score matching methodology. Specifically, we carried out a two-step approach as proposed in the literature [35]. First, a pairwise nearest neighbor (nn) matches with the replacement; and second, we estimated the treatment effect on the treated (ATET) on the matched population to account for the estimate’s variance [36, 37]. A more detailed description of this method is provided in Supplementary Material 1.

### Implementation and transaction cost

**Fig. 1:**
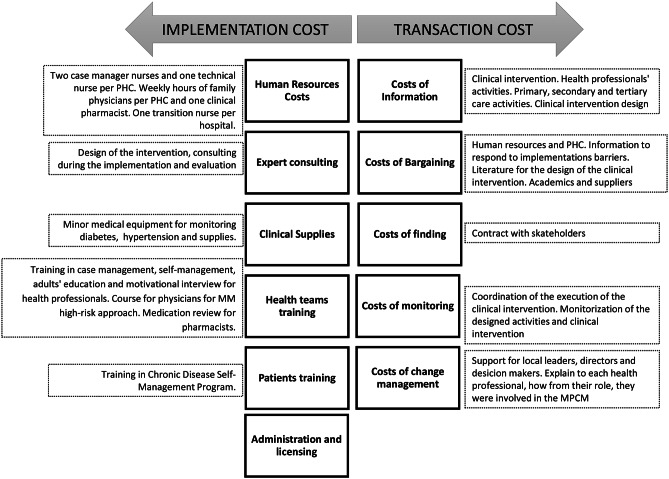
Implementation and transaction cost *PHC: primary care center; MM: Multimorbidity; MPCM: Multimorbidity patient-centered care model

Implementation costs were taken from the MPCM pilot study expenses and were annually registered in a database of the CISAUC at the Pontificia Universidad Catolica. We identified each item, adjusted them to the real execution period, and grouped them into 12 categories (Fig. 1). Transaction costs [38, 39] were identified exclusively for the team of the CISAUC that provided only external implementation support during the piloting period and did not perform clinical activities. They were classified as costs of information, costs of finding, costs of bargaining, costs of monitoring, and costs of change management support (Fig. 1). The methodology for valuation was the following. First, the activities of the four people in the CISAUC team were identified and grouped according to the types of transaction costs. Then each activity’s number of hours and periodicity was identified and valued. Finally, transaction costs were estimated for each category. Results of a previous evaluation of the implementation process were used as a secondary data source during the assignment of the costs [40].

## Results

The baseline characteristics show that 44% (20,359) of the patients were assigned to the intervention group, while the remaining 66% (26,340) were to the control group. The balance between the control and intervention groups is presented with a mean difference Test in Table 1. We found that the sample was significantly unbalanced in most covariates evaluated, revealing this routinely collected data’s selection bias.

**Table 1:** Mean difference test between control and intervention group

Variable	Control Group Average	Intervention Group Average	P-Value
Age	64,5	63,0	< 0.01
Men	33%	33%	> 0.1
FONASA A	23%	22%	< 0.01
FONASA B	47%	49%	< 0.01
FONASA C	12%	12%	> 0.1
FONASA D	18%	17%	< 0.05
Risk ACG 1	0%	2%	< 0.01
Risk ACG 2	18%	7%	< 0.01
Risk ACG 3	63%	61%	< 0.01
Risk ACG 4	19%	30%	< 0.01
N° of chronic diseases	4.2	5.4	< 0.01
N° previous hospitalizations	0.1	0.1	> 0.1
N° previous Ter ER consultants	0.5	0.5	< 0.05
N° previous PHC ER consultants	0.8	1.0	< 0.01
N° previous drugs	2.9	7.5	< 0.01
Previous year costs (USD)	1,171	1,231	< 0.05
Time follow-up (years)	1.20	1.10	< 0.01

* Ter ER: tertiary care emergency room consultants; PHC ER: primary care emergency room consultants

Direct costs for both groups are presented in Table 2, categorized by primary, secondary, or tertiary care. Furthermore, the Table also shows estimates for different items such as professional activities, drugs, emergency services, in hospital services. The expected cost for one patient under the MPCM model care was estimated as USD 901.23 and USD 933.30 for the standard model during the study’s average follow-up time. As expected, the highest proportion of this expenditure is at the tertiary level, i.e. hospital care. Indeed, almost 89% of this total cost is explained by hospital expenditure. In contrast, only 8% and 3% of the expenditure is due to primary and secondary care, respectively. On the other hand, medication costs account for 40% and 54% of the total cost registered in primary care for the control and intervention groups, respectively. At the secondary level, it represents only 20% and 11% of the reported cost.

**Table 2:** Estimation of Direct cost (USD) per patient during the intervention period

	Control Group		Intervention Group		Mean difference
	Mean (USD)	Standard Error (USD)	Mean (USD)	Standard Error (USD)	Treatment vs. control (USD)
**Total Costs**					
Total Costs	$933.30	$21.14	$901.23	$18.95	-$32.07
**Primary care**					
Total PHC Costs	$74.61	$0.66	$144.30	$2.40	$69.68
Physician visits	$23.16	$0.19	$35.78	$0.26	$12.61
Other professional consultation	$21.64	$0.23	$28.57	$0.28	$6.92
Emergency department visit	$1.06	$0.03	$0.90	$0.04	-$0.16
Drugs	$28.74	$0.42	$79.05	$2.27	$50.30
**Secondary care**					
Total secondary Costs	$24.93	$1.27	$27.91	$0.60	$2.98
Physician visits	$7.12	$0.16	$9.40	$0.19	$2.29
Other professional consultation	$12.55	$0.28	$15.98	$0.34	$3.42
Drugs	$5.26	$1.19	$2.52	$0.34	-$2.74
**Tertiary care**					
Total tertiary Costs	$833.76	$20.88	$729.03	$18.94	-$104.72
Emergency department visit	$3.41	$0.29	$2.69	$0.21	-$0.72
Hospitalization costs	$830.35	$20.85	$726.34	$18.92	-$104.00

*Change CLP (Chilean pesos) to USD at 1 USD to804 CLP. PHC: Primary health care

The effect of MPCM on direct costs post propensity score matching is presented in Table 3. Regarding aggregated costs, the MPCM decreases the total expected costs by 12% in the intervention compared to control patients. The effect of the level of care showed that costs are expected to grow for primary care (19% increase) and secondary level (7% increase), both statistically significant. In contrast, costs showed to be significantly lower at the hospital level, accounting for an expected 19% savings compared with standard care. Because of the higher costs at the hospital level compared to the other levels (see Table 1), these savings determine the estimates at the global level.

The higher cost of primary care is mainly due to higher expenditure on pharmacological treatments, followed by medical consultancies and other professional services. Interestingly, we found a significant decrease in costs related to emergency care at the primary level. At the secondary level, the greater cost is explained by higher expenses for non-medical consultants, with no statistically significant differences in other services or pharmaceuticals spent. At the hospital level, the effect is explained by hospitalizations, maintaining the same expenditure in emergency hospital services.

Table 3 also shows the effect of MPCM on different risk groups. The results show that the system incurs higher costs for high-risk patients in almost all items examined. It spends significantly more on primary care and secondary care. Indeed, the magnitude of the increase in professional services (medical and non-medical) increased significantly, which is expected from the model conceptualization. In tertiary care, our results show that the spending is not significantly higher than the standard.

In moderate-low-risk patients, our results also indicate significantly higher costs at the primary and secondary levels. It is worth noting that despite this cost increase, we observed a 73% decrease in costs related to pharmaceutical therapy at the secondary level, which is aligned with a 52% increase in the cost of this item in primary care. In other words, these results are consistent with transferring pharmaceutical care from the secondary to the primary care level. In addition, we observed a significant cost reduction of 17% due to consultants for primary care emergencies. This is consistent with the effort of more effective patient management by the health team, avoiding overload the emergency consultations. Finally, moderate-low-risk patients showed statistically significantly lower hospitalization or hospital emergency services costs. This result is consistent with preventing hospital services in moderate-low-risk patients, who should be managed mainly in primary care. The magnitudes observed, 28% and 18% cost reduction in emergency and hospitalizations, respectively, are quite important and explain an important part of the total impact of the MPCM on direct costs.

**Table 3:** Treatment effect estimation

Dependent Variable	Total Population	Moderate and low Risk Subgroup	High Risk Subgroup
Aggregated Costs			
All Care	-0.12 (0.03)***	-0.11 (0.04)***	0.16 (0.08)*
Primary Care	0.31 (0.02)***	0.34 (0.02)***	0.56 (0.04)***
Secondary Care	0.07 (0.04)**	-0.05 (0.04)	0.44 (0.15)***
Tertiary Care	-0.19 (0.04)***	-0.18 (0.04)***	0.07 (0.09)
Primary Care			
Medical consultants	0.21 (0.01)***	0.22 (0.01)***	0.49 (0.03)***
Non-medical consultants	0.1 (0.01)***	0.03 (0.01)**	0.76 (0.04)***
PH ER consultants	-0.27 (0.04)***	-0.17 (0.05)***	-0.23 (0.18)
Drug therapy	0.47 (0.03)***	0.52 (0.03)***	0.49 (0.11)***
Secondary Care			
Medical consultants	0.02 (0.03)	0.03 (0.03)	0.1 (0.11)
Non-medical consultants	0.18 (0.03)***	0.03 (0.03)	0.89 (0.08)***
Drug therapy	-0.33 (0.24)	-0.73 (0.27)***	-0.59 (0.29)**
Tertiary Care			
ER consultants	-0.08 (0.1)	-0.28 (0.08)***	0.23 (0.21)
Hospitalizations	-0.19 (0.04)***	-0.18 (0.04)***	0.07 (0.1)

() SE; *p-value < 0.1; **p-value < 0.05; ***p-value < 0.01; PHC ER: primary care emergency room consultants; ER: emergency room consultants

### Implementation costs

The total implementation cost for the three years was 1,838,767 USD (Table 4). It is observed that total costs increased by 46% between 2017 and 2018, which is associated with the piloted expansion to other PHCs and hospitals (2017, 4 PHCs and two hospitals; 2018, seven PHCs and three hospitals). The higher costs are observed in human resources and training, where the cost of human resources added to the PHC represents 63.5% of total implementation expenses. In contrast, the lowest costs were operational costs and workshop supplies. Other costs reported 0 expenses in 2019, such as clinical supplies and patient training.

**Table 4:** Implementation costs expressed in USD

Type of Cost	2017	2018	2019	TOTAL
Human resources at PHC	280,566	523,831	523,831	1,328,227
Human resources at secondary level	50,729	76,093	76,093	202,914
Expert consulting	27,354	5,968	4,849	38,171
Mobile, computers and internet	6,473	7,606	7,606	21,686
Transportation for home visits	25,364	29,000	14,500	68,865
Clinical Supplies	1,615	1,234	0	2,848
Workshops supplies	1,119	1,455	1,892	4,467
Office supplies	1,174	1,527	1,985	4,686
Other operational costs	827	1,075	1,397	3,298
Health teams training	20,351	9,243	46,128	75,722
Patient Training	16,510	18,650	0	35,161
Administration and licensing expenses	9,816	21,453	21,453	52,722
**Total**	**441,898**	**697,135**	**699,734**	**1,838,767**

### Transaction costs

The results show a total of 393,765 USD in transaction costs during three years (Table 5). An increase in costs of 28% between 2017 and 2018 is associated with the increase in the number of pilot centers mentioned above. Monitoring and reporting costs account for 68% of transaction costs. On the contrary, the lowest cost is the cost of Change Management, reporting 7% of the total cost.

**Table 5:** Transition costs expressed in USD

Type of Cost	2017	2018	2019	Total
Information	33,463	56,812	56,107	146,383
Finding	11,043	13,315	11,657	36,015
Bargaining	14,920	18,650	18,650	52,221
Monitoring	35,808	39,787	39,787	115,383
Change Management	7,294	14,589	21,883	43,766
**Total**	102,530	143,153	148,084	393,767

Finally, the routinely collected data allowed us to estimate the impact on the overall survival of individuals managed under the MPCM model compared to the standard care model. After the propensity score matching, our estimate was 0.77 (p-value 0.05; CI 95% 0.62–0.97), indicating an expected decrease of hazard reduction of 23% attributable to the MPCM care model.

## Discussion

The study aimed to examine the impact of the MPCM on direct costs, implementation costs, transition costs, and overall survival. The results suggest that the MPCM is associated with a 12% decrease in the total expected costs incurred by the healthcare system over the follow-up time. Furthermore, this reduction is mainly explained by the effect on tertiary care, which was estimated to reach an expected 19% cost reduction. In contrast, it shows a growth in secondary (7%) and primary care costs (31%). In terms of outcomes, our analysis indicates gains in survival attributed to the MPCM model compared to the standard model. These overall results are consistent with the effort of the MPCM to maintain and enhance patient care at the primary level.

Furthermore, the results indicate that the most important impact on cost reductions is explained by the effects observed in moderate-low risk populations. In this subgroup, we observed a significant cost reduction in hospitalizations and emergency care at the hospital level. Because hospitalization costs determine the highest proportion of the expected cost of one patient, this 18% cost reduction explains a large proportion of the overall cost reduction. It is worth noting that this is one of the main purposes of MPCM [], i.e. to reduce the use of health systems’ crucial resources, such as hospital services in patients with low-moderate-risk, who should be managed appropriately in primary care, decreasing demand for hospital services. In this context, we expected increasing costs in primary care. Another important result in this subgroup is the significant cost reduction in primary care emergency services. In other words, low-moderate patients are demanding fewer resources from emergency at primary care facilities, which are being translated to primary care management.

Our results observed for high-risk populations are also consistent with the MPCM purposes. In this group, we found higher primary and secondary care costs, which are expected from a model that introduces a higher frequency of care services. In terms of costs at the hospital level, we expected that MPCM would decrease those costs, but in the long run, because of the long-term effects of stabilizing diseases on the use of healthcare services. Nevertheless, during the follow-up period (mean 1,2 years), the effect of MPCM on the consumption of hospital services in the high-risk population was expected to be inelastic. In addition, the continuous increment in health services utilization and expenditure in hospital care to improve the performance of the health systems may also explain -at least partially- the absence of a significant cost reduction. A final element is the impact on overall survival in the MPCM group. If more patients remain alive, we expect the system to maintain expenditure on health services for those who otherwise would have died. In light of the results, we can conclude that MPCM maintained the costs in high-risk patients, decreased costs in low-moderate-risk patients, and improved their survival. These results become a strong body of evidence for policymaking.

Another important result is the 23% reduction in the hazard of death attributable to MPCM. These findings relate to the intervention purpose of having the PHC as the axis of chronic patient care, where tertiary care and mortality reduction reflect a greater control of multimorbidity achieved by the intervened patients. These findings are consistent with previous impact analyses of the MPCM on health system performance and health outcomes [28, 29]. We highlight that this estimate is more reliable than previous impact estimates due to the minimization of bias achieved after the propensity score matching.

A question that needs to be more widely described is how much it costs to implement complex changes such as MPCM. Bringing into real practice an intervention that involves the three levels of care in an already overwhelmed health system is a challenge that requires strong efforts in allocating resources [41]. Implementation and transaction costs reflect that complex changes do not occur alone and that investment is necessary to install the change, which is not linear. In this study, those costs represent 0.07% of the total budget of the SSMSO with a 1.5 million population coverage [42]. Although these costs cover 21% of the total primary care facilities of the SSMSO [43], it provides a clear idea about the magnitude of future implementations.

Furthermore, the costs associated with multimorbidity are often described as an expected costs for one or more diseases. The MPCM incorporates risk stratification of multimorbidity, based on Kayser Permanente [17], which allows us to estimate the expected cost of high, medium, and low-risk patients rather than the cost by disease, making this study quite unique in this sense. This categorization also offers an opportunity for a fairer allocation of resources to primary care adjusted by risk. This is particularly relevant in Chilean or similar contexts where the financing model for primary care follows a flat capitation model [44].

One of the study’s main strengths is the data’s characteristics. We had individual-based micro-costing data through a TDABC costing methodology, acknowledged as a powerful method for costing healthcare services [32]. Another strength is that it provides descriptive information about implementation and transaction costs. We are aware that this information depends highly on the jurisdiction where the model is implemented. However, our analysis can serve as a reference framework for the evaluations in other jurisdictions. For example, our transaction cost analysis may help to avoid underestimations through careful consideration of the items we included in this exercise.

Regarding limitations, we dealt with routinely collected data, which included some observations in the control group that did not incur any expense. Those patients probably opted to receive care in the private sector, where the information was unavailable. However, this was mitigated through propensity score matching. Another limitation of the study is its restricted economic perspective. Future studies may also include the impact of this model of care on out-of-pocket expenditure, which needs a collection of information from patients out of the scope of the health system records.

Finally, this study provides valuable evidence about the economic and health impact of the multimorbidity approach in healthcare provision. The MPCM has been shown to reduce overall health system costs and improve patients’ survival. Cost savings are explained by a significant cost reduction in hospital services, especially in low-moderate-risk patients. This evidence supports implementing a model like MPCM because it is consistent with the efficient use of public resources and aims to improve population health.

### Electronic supplementary material

Below is the link to the electronic supplementary material.

Supplementary Material 1

## Data Availability

All data generated or analyzed during this study are included in this published article and its supplementary information files.

## Abbreviations

CISAUC: Centro de Innovacion en Salud ANCORA UC
ATET: Treatment effect on the treated
MM: Multimorbidity
ACG: Adjusted Clinical Groups
TDABC: Time-Driven Activity-Based Costing
CESFAM: Family Health Center
MPCM: Multimorbidity Patient-Centered Care Model
FONASA: National Found of Health
PHC: Primary health care centers
SSMSO: Servicio Metropolitano Sur Oriente
