# Supplementary material for: Economic evaluation of a multimorbidity patient centered care model implemented in the Chilean public health system
Source: BMC Health Serv Res. 2023 Sep 29;23:1041. doi: 10.1186/s12913-023-09970-y (PMC10543850; doi:10.1186/s12913-023-09970-y)
Supplement: Supplementary file 1 — Supplementary Material 1 [file 12913_2023_9970_MOESM1_ESM.docx]

# Appendix 1.

Since the data does not stem from an experimental design, the raw analysis, namely the comparison of reported outcomes between treatment and control groups, cannot provide an unbiased estimation of the treatment effect due to potential systematic differences between these groups that were present before treatment assignment (selection bias). Even though regression models attempt to address this problem by controlling for certain variables, the issue of bias remains unsolved. To overcome this problem, we employed propensity score matching (PSM), a widely-used and validated method to balance the intervention and control groups in terms of their baseline covariates, increase their comparability, and reduce the selection bias (1, 2).

PSM compares the control and intervention groups by conditioning the probability of receiving treatment based on a set x of observable baseline covariates. Individuals with an equal probability of receiving treatment, known as clones, are statistically similar and, therefore, comparable (1, 2). By comparing these groups, it is possible to estimate the counterfactual outcome, which refers to what would have occurred if the treated individual did not receive the treatment, and vice versa if the non-treated individual had received it. Then, we can estimate the average treatment effect (ATE) or the average treatment effect over the treated (ATET) as a function of the outcomes recorded for both the intervention and control groups and the imputed counterfactuals (clones).

One of the significant advantages of PSM is that it enables us to summarize the impact of a set of covariates on receiving treatment into a single dimension called propensity score, which is not possible with multivariate matching methods like Mahalanobis distance matching (3). To assess the impact of MACEP intervention, we followed a two-step approach:

1. We executed a propensity score with a 1:1 nearest neighbour (nn) matching strategy.
2. We estimated the ATET using a gamma regression for cost outcomes and a Cox model for survival analysis. We chose the gamma regression since, as the dependent variable is cost, this model provides the best-fit model according to the AKAIKE (AIC) and Bayesian criteria (BIC) (4). Its beta coefficient can be interpreted as a semi-elasticity, which is convenient since we intended to evaluate the impact of the intervention on the direct costs of healthcare services.

A logit model that relates treatment assignment to a set of baseline covariates was estimated to estimate the propensity score or the probability of receiving treatment. The model is presented below:

$$P_{i}=\gamma X_{i}+\delta S_{i}+ {\theta Z}_{i} + {\text{Φ}C}_{i}+\Omega T_{i}+ \epsilon_{i}$$

Where the outcome *Pi* corresponds to a binary variable that describes whether the *i-*th subject is assigned to the intervention or control group; where *Xi* is a vector of individual sociodemographic covariates (age, sex, socioeconomic stratification); with *Si* as a vector of covariates that describe the health situation of an *i-*th individual (number of comorbidities, ACG risk, and E risk); *Zi* is a vector of covariates that report the number of healthcare resources utilized and total costs incurred by the *i-*th subject in the year before the start of the intervention; is the time under intervention or follow up time for non-treated individuals; and as the disturbance term.

After estimating the "distance" measure, we used the Stata package psmatch2 to perform matching (5). Since the number of control individuals is relatively low compared to the number of treated individuals, we selected a nearest-neighbour (nn) pair matching with a replacement strategy with a calliper of size 0.2 standard deviation to maximize resemblance among pair-matched individuals (3, 6). According to Austin et al., this calliper size can eliminate at least 98% of the selection bias (6). In the second step, in line with the nn matching implemented, we estimated an ATET ─ note as none of the treated individuals was discarded because of the calliper; the estimated effect remains as an ATET (7).

Regarding treatment effect estimation, for costs outcomes, to properly account for the variance of a treatment effect estimated by the linear model in the matched data, we performed the estimation of the standard error through the weighted least squares variance estimators described in the work of Hill et al. whereas, for survival outcomes, we use the variance estimator proposed in Austin et al. which account for the correlation among pair membership and the reuse of control units (i.e. replacement) (8, 9).

For the subgroup analysis, we carried out the stratification approach described by Green et al. to achieve covariate balance within each subgroup (10). This means that for each of the subgroups of interest, the two steps mentioned above were implemented ─ i.e. propensity score, matching and ATET estimation were performed within the subgroup.

Its ROC curve was used to analyze the predictive propensity score model's performance (Figure 1). The figure allows analyzing the degree of accuracy of the adjusted model to determine the allocation to treatment. An area under the curve of 0.82 was observed, which indicates that the predictive model is satisfactory.

**Figure 1.** ROC curve and performance of the predictive model used.

Once the propensity score or the probability of being assigned to the intervention group was estimated, a matching was made using the calliper technique.

References

1. ROSENBAUM PR, RUBIN DB. The central role of the propensity score in observational studies for causal effects. Biometrika. 1983;70(1):41-55.

2. Zhao QY, Luo JC, Su Y, Zhang YJ, Tu GW, Luo Z. Propensity score matching with R: conventional methods and new features. Ann Transl Med. 2021;9(9):812.

3. Stuart EA. Matching methods for causal inference: A review and a look forward. Stat Sci. 2010;25(1):1-21.

4. Blough DK, Ramsey SD. Using Generalized Linear Models to Assess Medical Care Costs. Health Services and Outcomes Research Methodology. 2000;1(2):185-202.

5. Leuven E, Sianesi B. PSMATCH2: Stata module to perform full Mahalanobis and propensity score matching, common support graphing, and covariate imbalance testing. 2018.

6. Austin PC. Optimal caliper widths for propensity-score matching when estimating differences in means and differences in proportions in observational studies. Pharm Stat. 2011;10(2):150-61.

7. Greifer N, Stuart EA. Choosing the estimand when matching or weighting in observational studies. arXiv preprint arXiv:210610577. 2021.

8. Austin PC, Cafri G. Variance estimation when using propensity-score matching with replacement with survival or time-to-event outcomes. Stat Med. 2020;39(11):1623-40.

9. Hill J, Reiter JP. Interval estimation for treatment effects using propensity score matching. Stat Med. 2006;25(13):2230-56.

10. Green KM, Stuart EA. Examining moderation analyses in propensity score methods: application to depression and substance use. Journal of consulting and clinical psychology. 2014;82(5):773.
